# Non-directional radial intercalation dominates deep cell behavior during zebrafish epiboly

**DOI:** 10.1242/bio.20134614

**Published:** 2013-06-03

**Authors:** Robert Bensch, Sungmin Song, Olaf Ronneberger, Wolfgang Driever

**Affiliations:** 1 Department of Computer Science, Albert-Ludwigs-University Freiburg, Georges-Koehler-Allee Geb 052, 79110 Freiburg, Germany; 2 BIOSS – Centre for Biological Signalling Studies, Albert-Ludwigs-University Freiburg, Schänzlestrasse 18, 79104 Freiburg, Germany; 3 Developmental Biology, Institute Biology I, Faculty of Biology, Albert-Ludwigs-University Freiburg, Hauptstrasse 1, 79104 Freiburg, Germany; 4 Spemann Graduate School of Biology and Medicine, Albert-Ludwigs-University Freiburg, 79104 Freiburg, Germany

**Keywords:** Gastrulation, Epiboly, Radial intercalation, Pou5f1

## Abstract

Epiboly is the first coordinated cell movement in most vertebrates and marks the onset of gastrulation. During zebrafish epiboly, enveloping layer (EVL) and deep cells spread over the vegetal yolk mass with a concomitant thinning of the deep cell layer. A prevailing model suggests that deep cell radial intercalations directed towards the EVL would drive deep cell epiboly. To test this model, we have globally recorded 3D cell trajectories for zebrafish blastomeres between sphere and 50% epiboly stages, and developed an image analysis framework to determine intercalation events, intercalation directionality, and migration speed for cells at specific positions within the embryo. This framework uses Voronoi diagrams to compute cell-to-cell contact areas, defines a feature-based spatio-temporal model for intercalation events and fits an anatomical coordinate system to the recorded datasets. We further investigate whether epiboly defects in MZ*spg* mutant embryos devoid of Pou5f1/Oct4 may be caused by changes in intercalation behavior. In wild-type and mutant embryos, intercalations orthogonal to the EVL occur with no directional bias towards or away from the EVL, suggesting that there are no directional cues that would direct intercalations towards the EVL. Further, we find that intercalation direction is independent of the previous intercalation history of individual deep cells, arguing against cues that would program specific intrinsic directed migration behaviors. Our data support a dynamic model in which deep cells during epiboly migrate into space opening between the EVL and the yolk syncytial layer. Genetic programs determining cell motility may control deep cell dynamic behavior and epiboly progress.

## Introduction

Vertebrate gastrulation combines three principle coordinated cell movements to establish the three germ layers and extend the embryonic axes (Keller, 2005; Leptin, 2005; Solnica-Krezel, 2005). Epiboly represents the spreading of embryonic cells over a vegetal yolk mass, resulting in ectoderm to cover the embryo. Emboly (ingression, involution, invagination) relocates the mesendodermal anlagen into the inner embryo. Convergence of vegetal and lateral cells to the dorsal axis and extension establish the long axis of the embryo. Epiboly, emboly and convergence each pose major challenges to the understanding of large-scale coordinated cell movements (Keller et al., 2008). Here, we investigate how blastoderm cells behave dynamically to achieve epiboly spreading over the yolk cell in the zebrafish embryo.

**Fig. 1.**
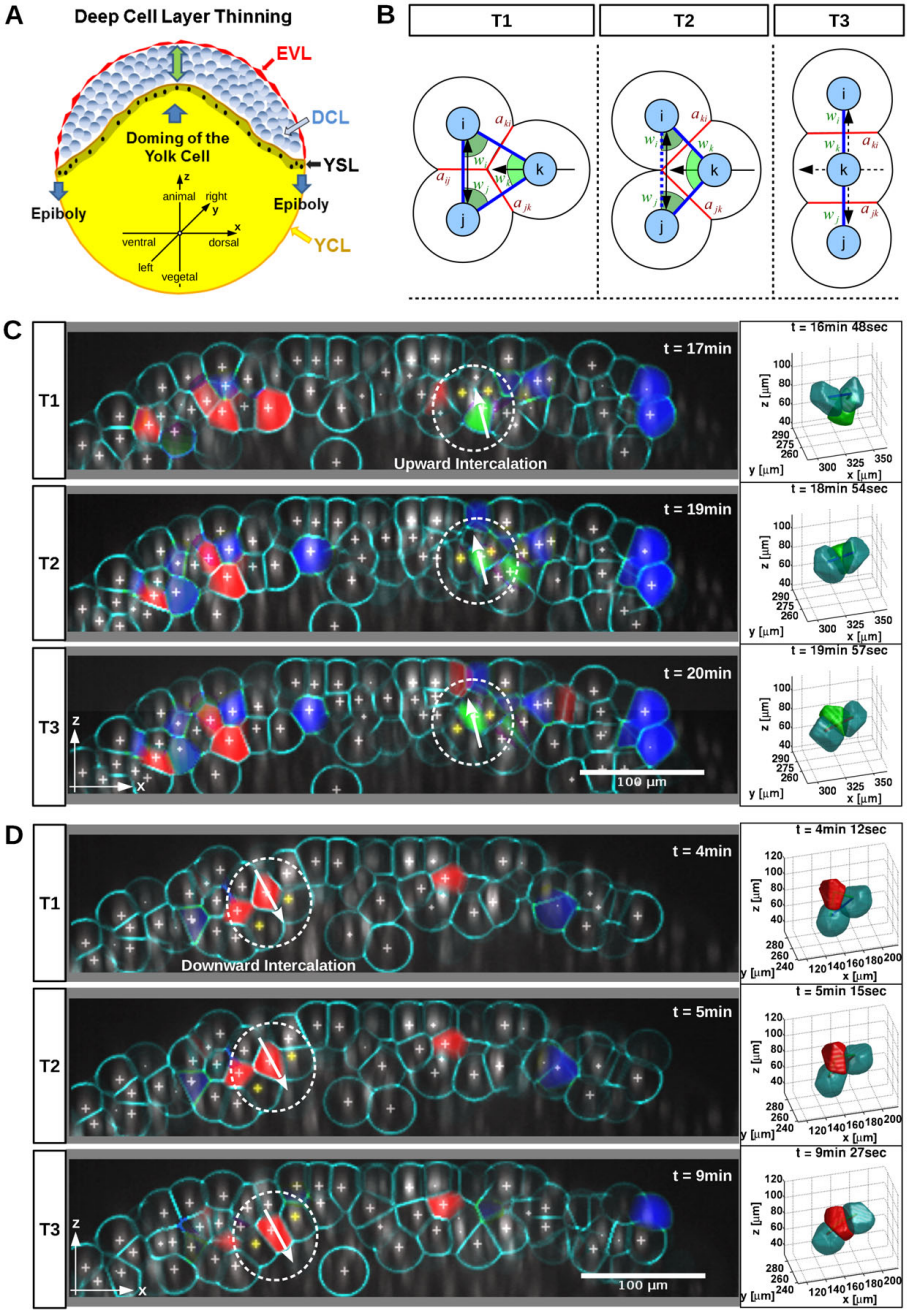
Automated detection of intercalating zebrafish blastomeres. (**A**) Schematic view of DCL thinning and epiboly during zebrafish early gastrulation with coordinate systems used. (**B**) Three-stage (T1 to T3) model of cell intercalation for point triple analysis. The cell located in the center (*k*) intercalates between the neighboring cells (*i* and *j*). Pairwise distances (blue), enclosing angles (green) and contact areas (red). (**C**,**D**) Computational detection and classification of radial intercalations from 3D time-lapse recording (supplementary material Movies 1, 2). Embryo stages: sphere to 50% epiboly. The rendering shows lateral views (animal pole at top) with raw nuclei fluorescence (grey), tracked nuclei positions (crosses) and calculated cell boundaries (cyan). Arrows indicate direction of cell migration. Upward (green), downward (red), and lateralward intercalations (blue) were detected along an 18 µm thick animal–vegetal oriented sheet transecting the embryo along its dorsoventral axis (shown here as y-projection representing 18 µm orthogonal to the z-stack). In the circled areas, a blastomere intercalates between two neighboring cells (yellow crosses) located in adjacent more exterior level (C) or in adjacent more interior level (D). These two groups of cells were separately rendered in 3D (right). Scale bars: 100 µm.

The zebrafish blastoderm is composed of enveloping (EVL) and deep cell layer (DCL). Beneath the blastoderm is the yolk syncytial layer (YSL), which contains yolk syncytial nuclei (YSN), and is continuous vegetalwards with the yolk cytoplasmic layer (YCL) (Warga and Kimmel, 1990; Solnica-Krezel and Driever, 1994; Kimmel et al., 1995). Epiboly of the zebrafish embryo is initiated by animalward doming of the yolk cell and vegetalward movement of YSL and vegetal EVL margin (Fig. 1A). Vegetalward spreading of the DCL results in thinning of the blastoderm layer at the animal pole and covering of the yolk mass. Several mechanisms have been shown to contribute to epiboly. In the yolk cell, long vegetal arrays of microtubules drive epiboly of the YSL (Strähle and Jesuthasan, 1993; Solnica-Krezel and Driever, 1994). It is unclear whether EVL cells actively spread or are passively drawn vegetally, although lamellipodia formation of EVL cells supports active migration behavior (Lachnit et al., 2008). DCL radial intercalation is regarded as the main driving force for deep cell epiboly by promoting thinning of the blastoderm layer (Keller, 1980; Warga and Kimmel, 1990). The DCL is comprised of six to eight levels of blastomeres during early gastrulation and becomes two to three levels thin by the end of gastrulation. As deep cells do not form strict cell layers but rearrange dynamically during migration (Fig. 1C,D; supplementary material Movies 1, 2), we use the term (depth) levels as opposed to cell layers inside DCL to denote virtual layers of average cell diameter width. A prevailing model suggests that differential adhesiveness due to differential radial expression of the adherens junction molecule E-cadherin (E-cad) between interior and exterior levels of epiblast cells (i.e. higher *cdh1* expression in the exterior levels than the interior levels) promotes unidirectional radial intercalation of epiblast cells from the interior to the exterior levels (Kane et al., 2005; Málaga-Trillo et al., 2009; Schepis et al., 2012). Indeed, loss of function phenotypes for E-cad show severe epiboly delay (Babb and Marrs, 2004; Kane et al., 2005; Shimizu et al., 2005). However, our recent study (Song et al., 2013) failed to identify a gradient of E-cad protein expression among blastomeres, and revealed that deep cell layer thinning is likely independent of E-cad expression gradients, but that E-cad mediated mechanisms controlling migration efficiency of blastomeres are crucial for deep cell epiboly.

Here, we analyze global deep cell migratory behavior in wild-type (WT) and MZ*spg* mutant embryos. MZ*spg* mutant embryos are deficient in the Pou5f1 (homolog of mammalian Oct4) transcription factor, and develop a severe delay in epiboly, while emboly proceeds similar to WT (Lunde et al., 2004; Reim and Brand, 2006; Lachnit et al., 2008). Quantification of radial and lateral intercalation dynamics of blastomeres reveals that radial intercalation is symmetric along the animal–vegetal axis of the embryo, which is not in line with the prevailing model of directed radial intercalation driving deep cell epiboly (Kane et al., 2005; Málaga-Trillo et al., 2009). Instead speed and migration efficiency of blastomeres appear to be crucial for the deep cell epiboly.

## Results

### Zebrafish gastrulation is initiated with symmetric radial intercalation of blastomeres

To investigate intercalation mechanism during zebrafish early gastrulation, we analyzed the trajectories of blastoderm cell nuclei in embryos labeled with NLS-tomato (Tomato fluorescent protein with nuclear localization signal) between sphere and 50% epiboly stage (Song et al., 2013). In this time window, epiboly leads to significant thinning of the animal cap blastoderm. Most nuclei were not continuously tracked throughout the complete time window, but trajectories capture nuclei motion only between two mitoses, as NLS-tomato is released into the cytoplasm during mitosis. These datasets allow extraction of the exact position of cell nuclei, but do not reveal cell boundaries. Putative individual cell regions and outer boundaries (“membranes”) were estimated by image analysis to allow the study and visualization of cell intercalations (see Materials and Methods). We preferred experimental nuclear over membrane label because knowledge of the exact positions of the nucleus as center of gravity of the cells facilitates analysis and classification of cell movements.

**Fig. 2.**
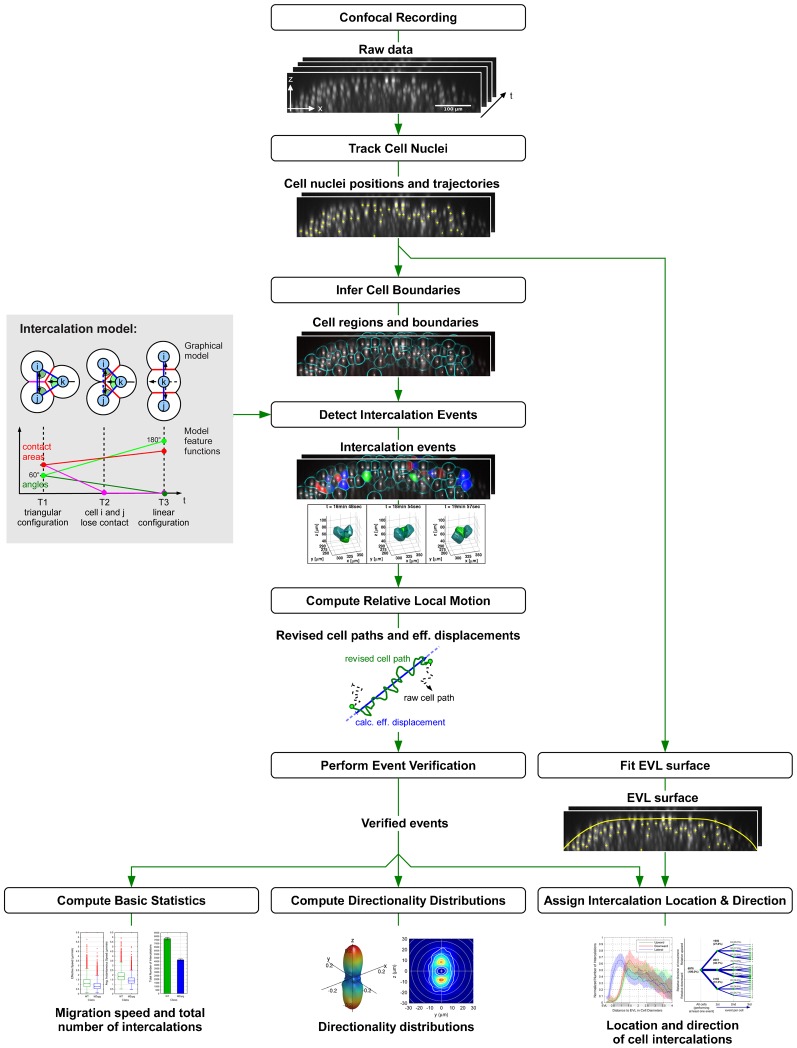
Workflow of image analysis. The workflow of image analysis is schematically presented: starting at the raw data input (top) the successive steps performing image analysis and data evaluations are presented. Details on the algorithms can be found in the Materials and Methods section with the same text headings. In the data flow intermediate results are illustrated that serve as input for the subsequent algorithm. Final results are presented at the bottom.

**Fig. 3.**
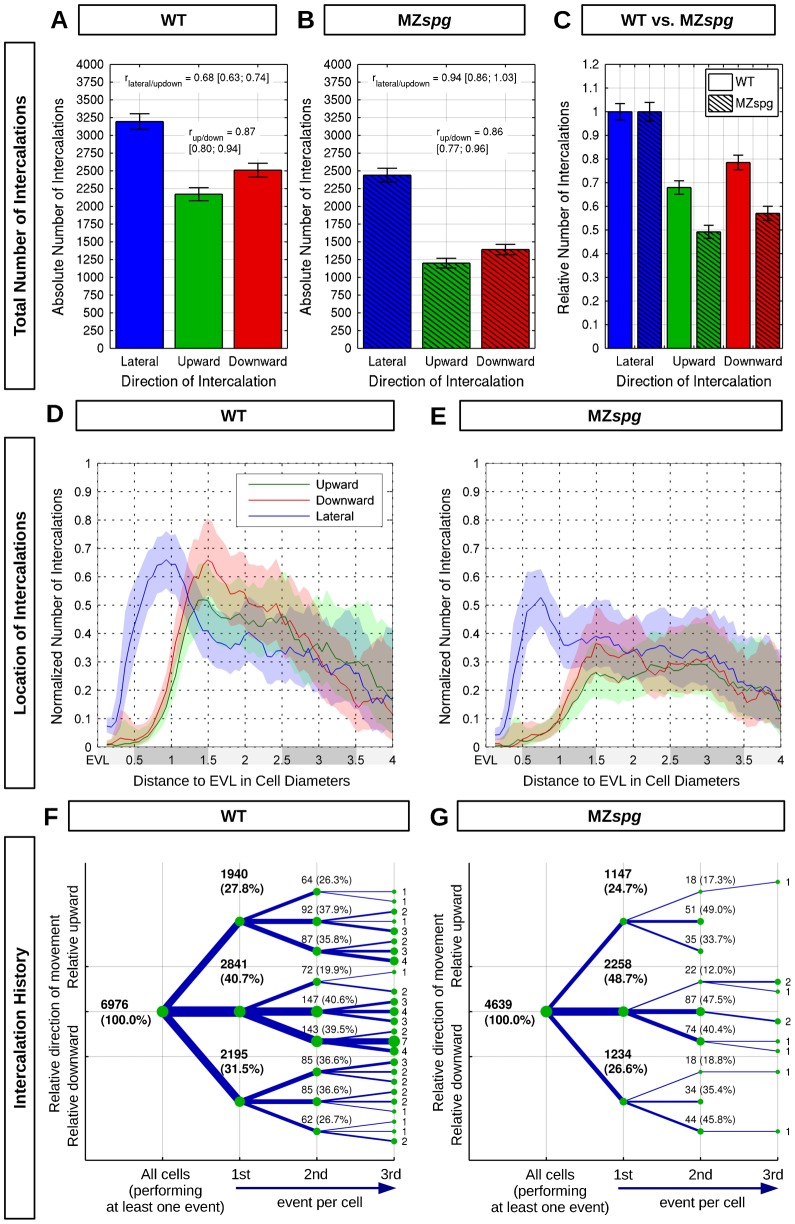
Quantification of radial and lateral intercalation events. (**A**,**B**) Absolute number of lateralward, upward, and downward intercalations in WT and MZ*spg* (summed over 6 embryos for WT and MZ*spg* each). Ratios between up- and downward, and between lateral and up-/downward intercalations are shown in each graph. (**C**) Relative number of upward or downward intercalations normalized to the number of lateralward intercalations. (**D**,**E**) Quantification of WT (D) and MZ*spg* (E) blastomeres performing upward, downward, or lateralward intercalations in each depth level. Depth levels (shaded grey along x-axis) were numbered and distance was measured starting from the EVL in vegetal direction. To be able to compare different depth levels, the absolute number of intercalations (summed over 6 embryos for WT and MZ*spg* each; supplementary material Fig. S1) was normalized by the total number of cells observed for each distance. The x-axis is truncated at 4.0, where the number of measured intercalations becomes too small to provide meaningful results. (**F**,**G**) Summarized intercalation history of all individual cells (sum over six embryos for each genotype). The graph presents up to three successive intercalations of individual blastomeres, indicating upward, downward, or lateralward directions. The root node (leftmost) denotes all cells performing the first intercalation event. The absolute number and relative fraction of intercalations is given at each node. Errors are given by 95% confidence intervals assuming Poisson noise.

The paradigm we used for cell intercalation analysis (see Materials and Methods) is shown in Fig. 1B. We determined upward (into more exterior level), downward (into more interior level), and lateralward (intra-level) intercalation events of blastomeres (Fig. 1C,D; supplementary material Movies 1–3). The workflow of the analysis is shown in Fig. 2, and the algorithms used in each step of the workflow described in Materials and Methods. To obtain a quantitative understanding of cell behavior during epiboly, we analyzed the total number of intercalations in each WT embryo dataset (Fig. 3A). Surprisingly, the total number of upward and downward intercalations was in the same range, with slightly more downward intercalations. This observation does not support the prevailing model that asymmetric radial intercalation of epiblast cells, i.e. inserting predominantly from an interior level into a more exterior level, drive DCL flattening (Kane et al., 2005). We further analyzed whether the epiboly delay phenotype of MZ*spg* embryos may correlate with different intercalation behavior. The total and relative number of upward and downward intercalations was significantly lower in MZ*spg* embryos than in WT, while the ratios between the upward and downward intercalations of blastomeres were balanced both in WT and MZ*spg* embryos (Fig. 3A–C). However, factors other than the total number of intercalations in a specific direction may affect epiboly progression, including directional bias in subsequent intercalations of individual cells, and dynamic aspects of cell movement. We further investigated both possibilities in detail.

### Location of intercalations and intercalation history

We next examined the number of intercalations along depth levels. Upward and downward intercalations were mainly distributed between the first and third DCL level in both WT and MZ*spg* embryos (Fig. 3D,E; supplementary material Fig. S1). However, in MZ*spg* embryos, the distribution of intercalations was shifted towards deeper levels compared to WT. This may be mainly due to significantly reduced thinning of the DCL in MZ*spg* embryos during the two-hour time window (supplementary material Movie 1). Therefore, in WT intercalations can only be detected in the first three levels at the end of the two-hour recording time, while in MZ*spg* embryos the deep cell layer is still thicker and intercalations can be detected in all levels. Furthermore, the first DCL depth level shows the highest number of lateralward intercalations both in WT and MZ*spg* embryos. This is reminiscent of previous reports that cells in the first DCL level are connected to EVL by E-cad mediated adherens junctions, suggesting them to be dragged by EVL during epiboly (Shimizu et al., 2005). However, cells in the first DCL level frequently moved back into the deeper levels both in WT and MZ*spg* embryos during the two-hour observations, suggesting that adherens junctions were dissociated.

We next measured the number of blastomeres performing subsequent intercalation events towards the three different directions (up-, down-, and lateralward; Fig. 3F,G). During the first intercalation event WT blastomeres performed 28% upward, 41% lateralward, and 31% downward intercalations (Fig. 3F). In MZ*spg* embryos 25% upward, 49% lateralward, and 26% downward intercalations were measured (Fig. 3G). Following their first intercalation many blastomeres performed a second and third intercalation in any of those three directions. These data clearly indicate that most blastomeres have the ability to perform subsequent intercalations in any direction with similar directionality distribution as in previous events. Supplementary material Fig. S2 shows the intercalation history of cells grouped by the depth level position of their first intercalation event, confirming that there is no directional bias. Similar observations were made for MZ*spg* mutant embryos. In summary, cells have no directional intercalation bias based on previous intercalation events, which we interpret to exclude that some extrinsic signal may initiate irreversible cell-intrinsic processes that would determine directionality.

### Quantification of intercalation directionality

**Fig. 4.**
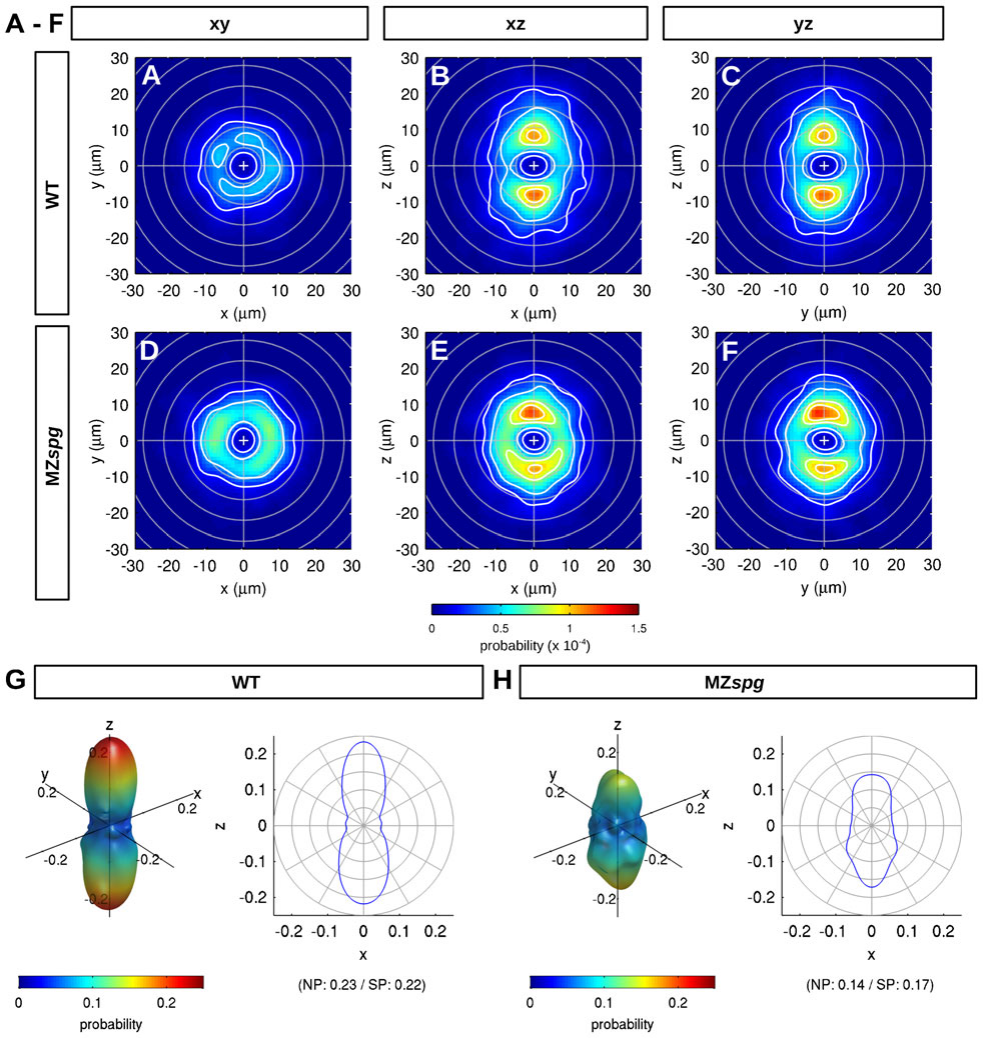
Motion directionality of intercalation events. (**A–F**) Average motion directionality analyzed for WT (A–C) and MZ*spg* (D–F) mbryos. The occurrence probability for an intercalation with certain migration direction and displacement is indicated by color. Isocontours (white) denote lines of equal probability. Cross-sections of 3D directionality distributions are given: x–y plane (A,D), perpendicular to animal–vegetal axis, x–z plane (B,E) and y–z plane (C,F), perpendicular to left–right and dorsoventral axis, respectively. (**G**,**H**) 3D reconstruction of the average motion directionality for WT (G) and MZ*spg* (H) embryos (modeled by spherical harmonics of degree *l* = 0…10). (Left) 3D rendering visualizing occurrence probability of intercalation directions using both color (blue = low and red = high probability) and shape (extension in each direction corresponds to probability). (Right) 2D plot visualizing occurrence probability of intercalation directions averaged along the latitudes. (*P*<0.01; *n* = 6 embryos each WT and MZ*spg*).

To investigate differences in global radial intercalation rates, we compared average motion directionality and cell speed during intercalation events between WT and MZ*spg* embryos. Blastomeres intercalate laterally in a rotationally symmetric distribution around the animal–vegetal axis, both in WT and MZ*spg* embryos (Fig. 4). In contrast, radial intercalation directions are distributed polarized in animal as well as vegetal direction along the animal–vegetal axis both in WT and MZ*spg* embryos (Fig. 4B,C,G and Fig. 4E,F,H). Strikingly, 3D directionality distributions indicate a stronger polarization in animal–vegetal direction in WT embryos than in MZ*spg* embryos, represented by the significantly higher relative number of intercalations toward the animal and vegetal pole of the WT embryos (North Pole (NP): 
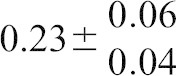
; South Pole (SP): 
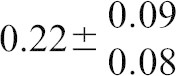
) than in MZ*spg* (NP: 
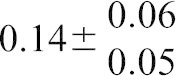
; SP: 
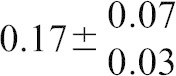
) (Fig. 4G,H). Accordingly, the ratio between lateralward intercalations and up- or down- intercalations is higher in MZ*spg* embryos (0.94) than in WT (0.68) (Fig. 3A–C). These data reveal that in MZ*spg* mutant embryos the directionality of total intercalation events may be affected by modulation of motility of blastomeres (Song et al., 2013), but may also be caused by a delay in the onset of epiboly.

### Analysis of spatial and temporal patterns of intercalation

**Fig. 5.**
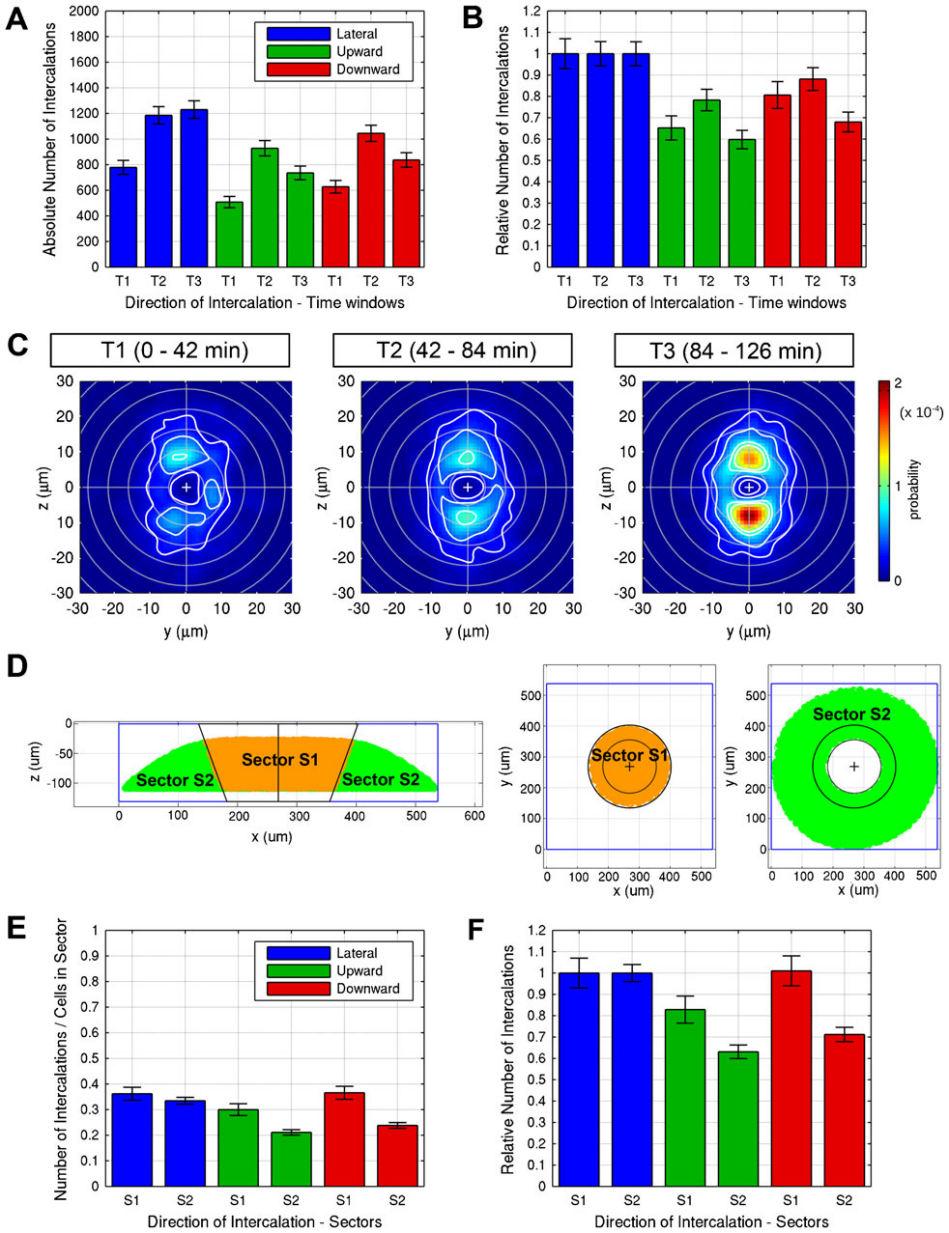
Analysis of spatial and temporal patterns of intercalation. (**A**,**B**) Absolute number (A) of lateralward, upward, and downward intercalations in WT for the time windows T1 (0–42 min), T2 (42–84 min), and T3 (84–126 min). The data are summed over 6 embryos each. (B) Relative number of upward or downward intercalations in each time window normalized to the number of lateralward intercalations. (**C**) Average motion directionality analyzed for WT embryos for each of the time windows T1 to T3. The occurrence probability for an intercalation with certain migration direction and displacement is indicated by color. Isocontours (white) denote lines of equal probability. Cross-sections of 3D directionality distributions are given for the y–z plane. (**D**) To analyze potential differences in intercalation behavior between an inner sector located centrally at the animal pole and an outer sector encompassing more marginal and vegetal cells, the 3D space of the image data stack was separated into an inner sector S1 (orange) and an outer sector S2 (green), visualized in lateral (left) and animal pole views (right).(**E**,**F**) Number (E) of lateralward, upward, and downward intercalations in WT for the sectors S1 and S2 normalized by the number of cells for each sector. The data are summed over 6 embryos each. (F) Relative number of upward or downward intercalations in each sector normalized to the number of lateralward intercalations.

The time window of our analysis spans from sphere to 50% epiboly stages, a period during which different forces may contribute to epiboly. Doming of the yolk may affect cells in a different manner as compared to progress of epiboly between 30% and 50% epiboly. Therefore, we reanalyzed our data in three time windows covering the first 42 minutes roughly equivalent to doming, the time from 42 to 84 minutes equivalent to early epiboly stages, and from 84 to 126 minutes equivalent to 30–50% epiboly. The precision in determining developmental stages is estimated to be in the range of ten minutes between different embryo recordings, which argued against analysis of even shorter time windows. We find that during doming, in WT there are significantly less total intercalations (Fig. 5A), but the ratio between lateral, up- and downward intercalations is not much different from the later two time windows (Fig. 5B). We also compared the total number of intercalations between WT and MZ*spg* in each time window, and find that while there are significantly less intercalations in MZ*spg* during time windows T1 and T2, the intercalation rate is similar between both genotypes in time window T3 (supplementary material Fig. S4B). The later onset of epiboly in MZ*spg* thus contributes to the differences in intercalation behavior between the genotypes.

We also analyzed the depth distribution of radial intercalations in WT in each time window (supplementary material Fig. S4A), and found that the depth profile changes slightly from the first time window, when up, down and lateral intercalations appear at similar rates at depth levels from 1.5 to 3 cells distant from EVL, to the last time window, when the normalized number of intercalations in these three directions is higher in depth layers closer to the EVL than in deeper layers.

We further analyzed whether cells in the animal central portion of the blastoderm at the animal pole may behave differently from those located more towards the vegetal margin. We defined an inner sector S1 representing the central animal cells, and an outer sector S2 representing the more vegetally located and marginal cells (Fig. 5D). Given that S1 contained less cells than S2, we normalized the number of intercalations in each sector to the cell number. The inner sector S1 has higher up- and downward intercalation rates, with the downward intercalations nearly as strong as the lateral intercalations (Fig. 5E,F). In sector S2 the number of lateral intercalations significantly exceeds the downward intercalations. When analyzing the depth distribution of intercalation rates, inner sector S1 cells have a similar distribution of intercalation directions from layers 1.5 through 3.5, while in sector S2 the normalized number of intercalations per cell decreases in deeper layers (supplementary material Fig. S4C).

### Quantification of radial intercalation dynamics

**Fig. 6.**
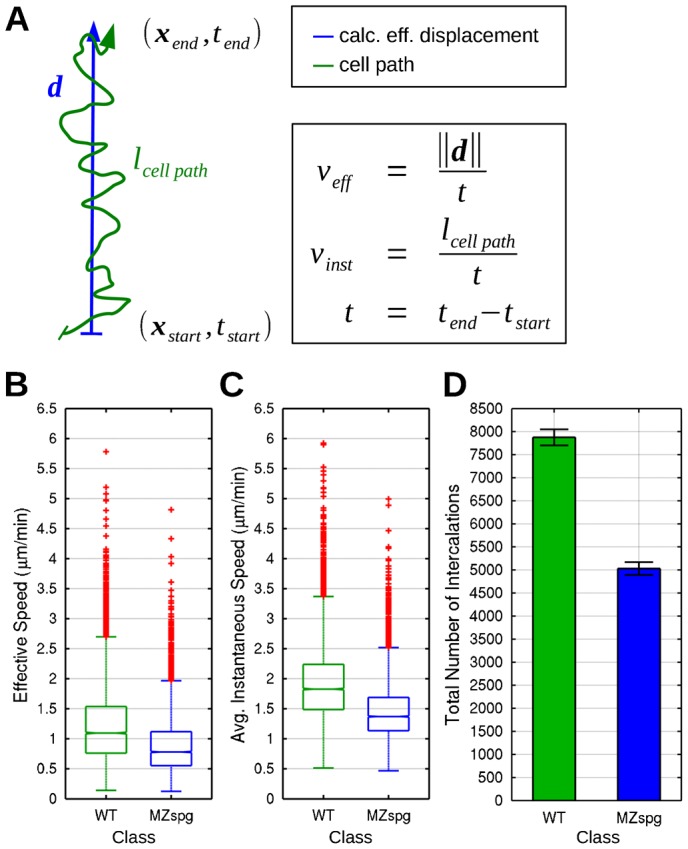
Migration speed of intercalating cells. (**A**–**C**) Radial intercalation dynamics. The effective and average instantaneous speeds of intercalating blastomeres were quantified. (A) Schematic drawing of cell path (green) and calculated effective displacement (blue) during intercalation. Calculation and comparison of effective (B) and average instantaneous speed (C) for WT and MZ*spg* embryos (*P*<0.05; *n* = 6 embryos each WT and MZ*spg*; Standard MATLAB boxplots). (**D**) Quantification of the total absolute number of intercalations for WT and MZ*spg* embryos (*P*<0.05; summed over *n* = 6 embryos each WT and MZ*spg*). Error bars show 95% confidence intervals assuming Poisson noise.

Given the importance of effective movement for intercalations, we investigated the influence of loss of Pou5f1 activity on the dynamics of cell behavior during radial intercalation by measuring effective speed and average instantaneous speed of blastomeres undergoing intercalations during early gastrulation (Fig. 6A). Both median effective and median average instantaneous speed was significantly higher in WT embryos than in MZ*spg* embryos (Fig. 6B,C), suggesting Pou5f1-dependent mechanisms are important for control of migration speed of intercalating blastomeres. Supplemental to the analysis of migration speed during intercalation, measured absolute effective displacements and cell path lengths are given in supplementary material Fig. S5. We also determined the total number of intercalations, which was significantly higher in WT embryos than in MZ*spg* (Fig. 6D). These data together indicate that Pou5f1 affects the total number of intercalations of blastomeres by controlling cell motility, especially migration speed of cells.

## Discussion

Gastrulation is an excellent model to study mechanisms controlling coordinated movements of large numbers of cells. However, even for the earliest gastrulation movement, epiboly, there is little understanding of the mechanisms that regulate this movement spatially and temporally throughout the embryo. Here, we used the zebrafish for a detailed analysis and description of intercalation cell behavior during the first two hours of zebrafish gastrulation, from sphere stage to doming of the yolk and epibolic spreading of cells up to 50% epiboly. We aimed to record most cell movements based on the position of their cell nuclei in one coherent data stack, which limited our analysis to about 50% epiboly stage, as we were not able to image throughout the embryo with a confocal laser scanning microscope at later stages. While other techniques have enabled whole embryo documentation (Keller et al., 2008) and analysis of surface movement of cells, such data have not been analyzed for cell behavior orthogonal to the surface, which is essential for analysis of radial cell intercalations. Our global cell intercalation study therefore focused on early epiboly stages, while previous analyses of small regions of the embryo investigated cell behavior at late epiboly stages from 70 to 90% epiboly (Kane et al., 2005).

We used a mathematical three point model to analyze intercalation cell behavior, which enabled us to apply image analysis algorithms to automatically detect and characterize cell intercalations throughout the 3D data volume and the two hour time lapse recording. The results showed that upward, downward and lateral intercalations occur throughout the deep cell layers, and surprisingly revealed similar rates of up- and downward intercalations with regard to the EVL surface, which argues against intercalations directed towards the EVL to be the major force to shape spreading and thinning of deep cells during epiboly. Observing individual cells also revealed no long-term bias in intercalation directionality: following a first intercalation, cells that performed a second intercalation did not show any bias in up-, down-, or lateralward direction. Thus, it appears that cells during early epiboly do not appear to become intriniscally programmed to intercalate in a defined direction only.

We also investigated changes in cell behavior in three time windows for doming, early epiboly and 30–50% epiboly. We found that in WT embryos, the intercalation directionality is not very prominent during doming, but a clear animal–vegetal directional bias is established during early epiboly, with similar upward versus downward intercalation distribution along this axis. We also found that the depth distribution of intercalations changes as epiboly progresses: while during dome stage up-, downward and lateral intercalations appear at similar low frequencies in layers one to three cell diameters away from the EVL, during mid-epiboly a profile is established in which the frequency of intercalating cells is higher in the upper cell layers compared to deeper ones.

The cell intercalation data raise the question whether they are sufficient to explain blastoderm thinning and epibolic spreading towards the vegetal pole. First, it appears counter-intuitive that a high number of both up- and downward intercalations has been detected, because if they occur in the same layers, this would effectively eliminate any net effect on expanding the DCL. However, the depth profile of intercalation events normalized to cell numbers in each depth layer reveals that from dome stage, when the profile is even, a gradient of intercalation rates establishes with higher intercalation rates in deep cell layer one to two as compared to layers three to four. Together with the slightly higher propensity for downward intercalations, this may effect a net redistribution of cells by intercalation to promote thinning and spreading of the DCL. We attempted to evaluate quantitatively the number of cells exiting the inner sector S1 in comparison to the number of radial intercalations (supplementary material Fig. S6). To visualize temporal changes we performed the analysis in eight 15 minute time windows, and also determined the ratio of the number of cells leaving the sector S1 and the number of radial intercalation events. Supplementary material Fig. S6 reveals that the number of cells leaving sector S1 trails behind the number of radial intercalations, until shortly before 50% epiboly, when exiting cells and radial intercalations occur at a ratio of approximately one. This analysis would be consistent with up- and downward intercalations partially compensating each other during doming and early epiboly, while intercalations may drive epiboly more effectively when the directionality (Fig. 5C) and the steeper radial profile of intercalations (supplementary material Fig. S4A) are established at 50% epiboly. However, it has been impossible for us to exactly quantitate the contribution of intercalation events to epibolic spreading, because in the time window analyzed, also the number of cells approximately doubles as the asynchronous thirteenth cell cycle progresses, and towards the end of the recording cells also have only about half of the volume each as compared to sphere stage.

Two models have been put forward about the forces that drive DCL epiboly (Keller et al., 2003). In one model, radial intercalation of deep cells is the driving force to spread the DCL. Here, directional cues would have to orient the intercalation behavior. Adhesion gradients, specifically of E-cad, have been proposed to direct radial intercalation during late epiboly to predominantly occur in the direction towards the EVL (Kane et al., 2005). Our analysis reveals that such a directional intercalation cannot be detected during early epiboly stages. In the second model (Keller et al., 2003), radial intercalation may be a more indirect effect of migrational spreading over yolk cell or EVL surfaces. For zebrafish, in this model epiboly of the YSL and EVL would open a space into which deep cells migrate. The prevalence of intercalation orthogonal to the EVL surface observed here may be caused by this type of intercalation effectively filling space opened up by EVL/YSL epiboly. Here, the dynamics and effectiveness of deep cell migration would be crucial for DCL epiboly progress, which is confirmed by our measurements. This is also consistent with the changes observed in MZ*spg* mutant embryos, in which E-cad trafficking and adhesion is affected in a way to reduce effective cell movements (Song et al., 2013). Our study provides a new approach to investigate dynamic behavior and intercalations of individual cells within a tissue during embryo development. This method may also be exploited in other fields such as cancer research to quantify epithelial–mesenchymal transitions *in vivo*.

## Materials and Methods

### Zebrafish maintenance and image acquisition

AB/TL strain was used as WT control. For embryos devoid of Pou5f1 function maternal and zygotic *spg^m793^* mutants (MZ*spg^m793^*) were used. 3D time-lapse recording of global blastomere migration was performed by Song et al. (Song et al., 2013). All nuclei were labeled by microinjection of *nls-tomato* mRNA (50 pg) at the one-cell stage. The 3D time-lapse stacks were recorded using a LSM5 Live Duo confocal microscope (Zeiss, Jena) with a Zeiss LD LCI Plan-Apochromat 25×/0.8 objective lens. Laser wavelength: 532 nm, filter: BP 560–675, z stack depth: 109.4 µm, duration: 126 min with 1.05 min intervals.

### Image quantification

All analyses presented here depend on the position of the nuclei recorded in the primary data. However, virtual cell boundaries are used to define neighborhoods of cells and provide features for the detection of cell intercalations. The full image analysis pipeline is described in the following paragraphs and depicted in Fig. 2.

### Track cell nuclei

Trajectories of labeled cell nuclei were obtained by applying the track spot tool of Imaris software (Bitplane, v7.3) using following parameters: estimated diameter: 7 µm, background subtraction, tracking algorithm: Brownian motion, MaxDistance: 12 µm, MaxGapSize: 2.

### Infer cell boundaries

Putative individual cell regions and outer boundaries (“membranes”) were estimated by a 3D Voronoi diagram using the nuclei positions as seeds. The maximal size of a cell was limited to a sphere with 20 µm radius to obtain reasonable cell regions at the borders. With this approach, the size of bordering cells (especially the flat EVL cells) is overestimated in outward direction. However, inner cell boundaries are estimated well and the conducted analysis described in the following is not affected.

### Intercalation model

Cell intercalations were modeled based on the intercalation model depicted in Fig. 1B and Fig. 2. This model describes an intercalation in terms of the angles (*w_i_*, *w_j_* and *w_k_*) and contact areas (*a_ij_*, *a_jk_* and *a_ki_*) of three cells (*i*, *j*, and *k*). The three cells start in a triangular configuration (time point T1) and end in a linear configuration (time point T3). Time point T2 models the point when cells *i* and *j* lose their contact. The start and end values of the six features for an ideal intercalation were manually defined by inspecting several clear intercalation events in the data set. Furthermore we assume a linear transition for all features from the start to the end values, except for *a_ij_*, which drops to zero between T1 and T2 and stays zero between T2 and T3.

### Detect intercalation events

For each cell the above described intercalation model was fitted to the trajectories using every possible combination of start time, end time and neighboring cells. The mean squared errors from the fit and the deviation of the fitted parameters to the ideal parameters were linearly combined (using manually specified weights) to a single error *d* and converted to a score *s* with range [0…1] by taking the exponential of the negated error, *s* = exp(*−d*). From these spatiotemporal scores local maxima in time are selected as events and overlapping events are joined to a single event. Finally detected intercalation events with low reliability (score <0.85) were discarded. The exact value of this threshold is not critical for the further analysis, as there is a smooth transition between intercalations and non-intercalations, and only relative numbers are considered.

### Compute relative local motion

The motion of intercalating cells relative to the local tissue was computed by successive temporal registration of local groups of cells. This compensates for translational motion from both global motion and local growth motion of the tissue. The resulting relative raw cell path is depicted schematically in supplementary material Fig. S5A (dashed black). From this raw cell path we compute the main motion direction, the calculated effective displacement and the revised cell path (supplementary material Fig. S5A). The main motion direction is found by principle component analysis (PCA) and represents the line of best fit with respect to the raw cell path. The extremal points in this main direction are used to refine the start and end point and the time window of the intercalation event, resulting in the calculated effective displacement and the revised cell path.

### Perform event verification

To reduce false positive detections, a subsequent verification step discards events that are not likely to be intercalations. First, intercalating cells are likely to show an effective displacement in the range of a cell diameter (cf. Fig. 1B) and second, intercalating cells are likely to perform a rather directed motion through neighboring cells. Therefore, we discarded events with less than 6 µm absolute effective displacement and directedness *r*_dir_ less than 0.85, with *r*_dir_ being the ratio of the first eigenvalue to the sum of eigenvalues obtained from principle component analysis (PCA) of the revised cell path.

### Compute basic statistics

Form the verified intercalation events, some basic statistics are plotted. The calculated effective displacement is used for the absolute effective displacement (supplementary material Fig. S5B) and effective speed (Fig. 6B). The revised cell path is used for quantifying cell path length (supplementary material Fig. S5C) and average instantaneous speed (Fig. 6C).

### Compute directionality distributions

3D directionality distributions were computed by density estimation, i.e. by accumulating measurements into a single 3D distribution with the starting point of the event centered at the origin (Fig. 4A–F). High density peaks are obtained when many events show similar direction. To describe isotropy and polarity, the 3D directionality distributions were projected onto the unit sphere and modeled by spherical harmonics (Rose, 1995) basis functions *Y*_*lm*_ (supplementary material Fig. S3A). The resulting expansion coefficients *c_lm_* with zero order (*m* = 0) in every band *l* (i.e. *c_l_*_0_) describe the distribution of the signal from North to South pole (averaged along the latitudes). For the present application, especially the second coefficient *c*_20_ is important (supplementary material Fig. S3A,B). It is positive, if the signal is located at the poles, negative if the signal is located at the equator, and zero, if the signal is homogenous.

### Fit EVL surface

An anatomical embryo coordinate system was defined by fitting a smooth surface to EVL cell nuclei for each time point.

### Assign intercalation location and direction

The fitted EVL surface is used as anatomical reference. The direction of the intercalation and the directions of successive intercalations were obtained by measuring the angle of the calculated effective displacement (Fig. 6A, blue) to the surface normal. The locations of the intercalations were obtained by measuring the distance to this surface. For representation the distances are discretized into cell diameters (Fig. 3D,E; supplementary material Fig. S1 and Fig. S4A,C). The reference cell diameter was measured by the statistics of nearest neighbor distances for all cells and time steps in all datasets. We used the median value *d* = 16.1095 µm as an estimate for the reference cell diameter. Directions were classified into “lateral”, if the displacement component in direction to the EVL was within ±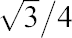 *d* (∼7 µm) and “upward” or “downward” otherwise.

### Statistical analysis

Statistical significance in terms of directionality of intercalation in WT and MZ*spg* (Fig. 4) was evaluated using the non-parametric Wilcoxon rank sum test. The test was based on the expansion coefficient *c*_20_ describing isotropy and polarity (supplementary material Fig. S3, *n* = 6 samples per class). For the description of WT and MZ*spg*, measurements were averaged (Fig. 4, Fig. 5C) or summed over 6 datasets per class (Fig. 3, Fig. 5A,B, Fig. 5E,F, Fig. 6D; supplementary material Figs S1, S2, S4). Standard MATLAB boxplots were used to plot class distributions (Fig. 6B,C; supplementary material Fig. S5B–D): the central mark is the median, the edges of the box are the 25^th^ and 75^th^ percentiles, the whiskers extend to the most extreme data points not considering outliers, and outliers are plotted individually (red crosses). The medians are significantly different at the 5% significance level if their comparison intervals (notches) do not overlap. In Fig. 3A–E, Fig. 5A,B, Fig. 5E,F, Fig. 6D, supplementary material Figs S1 and S4 the error is given by the 95% confidence intervals assuming Poisson noise when counting intercalation events.

All computations and statistical evaluations were performed in MATLAB (The MathWorks Inc.) or C++.

## Supplementary Material

Supplementary Material
