# Supplementary material for: Non-directional radial intercalation dominates deep cell behavior during zebrafish epiboly
Source: Biol Open. 2013 Jun 3;2(8):845–54. doi: 10.1242/bio.20134614 (PMC3744077; doi:10.1242/bio.20134614)
Supplement: Supplementary Material [file supp_2_8_845__index.html]

Non-directional radial intercalation dominates deep cell behavior during zebrafish epiboly — Supplementary Material 

# Non-directional radial intercalation dominates deep cell behavior during zebrafish epiboly

## 

**Files in this Data Supplement:**

- Supplementary Material - Robert Bensch et al. doi: 10.1242/bio.20134614
- Movie 1 - **Movie 1. Radial intercalation events during blastomere movement.** Global blastomere migration analyzed by computational detection and classification of radial intercalation events from two hour 3D time-lapse recording of WT (**A–C**) and MZ*spg* (**D–F**) embryos. Embryo stages: sphere to 50% epiboly. Confocal stacks (109 µm) were recorded from animal pole EVL into the margin of blastoderm over the doming yolk of the embryos. The renderings show lateral views (animal pole to the top) with raw nuclei fluorescence (grey), tracked nuclei positions (crosses) and calculated cell boundaries (cyan). Upward intercalations into more exterior levels (green), downward intercalations into more interior levels (red), and lateralward intra-level intercalations (blue) were detected along an 18 µm thick animal–vegetal oriented sheet transecting the embryo along its dorsoventral axis. (A,D) All intercalation events. (B,E) Upward and lateralward intercalations. (C,F) Downward and lateralward intercalations. Scale bar: 100 µm.
- Movie 2 - **Movie 2. Examples for intercalation events within a group of blastomeres.** Rendering of individual upward and downward intercalation events from 3D time-lapse recording of WT embryo (supplementary material Movie 1A–C). (**C**) Lateral view rendering (animal pole to the top) with raw nuclei fluorescence (grey), tracked nuclei positions (crosses) and calculated cell boundaries (cyan). Upward intercalations into more exterior levels (green), downward intercalations into more interior levels (red), and lateralward intra-level intercalations (blue) were detected along an 18 µm thick animal–vegetal oriented sheet transecting the embryo along its dorsoventral axis. Downward intercalation is shown exemplarily inside the red rectangle during the first 10 minutes of the movie. Upward intercalation is shown exemplarily inside the green rectangle during the following 20 minutes of the movie. (**A**,**B**) 3D rendering of corresponding individual cells performing upward or downward intercalation within a group of blastomeres. Scale bar: 100 µm.
- Movie 3 - **Movie 3. Directional analysis of migrating blastomeres.** The calculated effective displacement (Fig. 6A) of all detected intercalations from WT and MZ*spg* embryos from two hour 3D time-lapse recording (supplementary material Movie 1) were visualized. Colors indicate the classified direction (upward: green, downward: red, and lateralward: blue). Three different views are shown: x–y plane (animal top view of the embryo), x–z plane and y–z plane (lateral views of the embryo). Motion direction is indicated in each view additionally by different symbols (lateralward: square; top view upward: circle, downward: cross; side views up-/downward: up-/downward pointing triangle). Point traces are shown for the past 5 minutes preceding each frame only.
