# Supplementary material for: Non-directional radial intercalation dominates deep cell behavior during zebrafish epiboly
Source: Biol Open. 2013 Jun 3;2(8):845–54. doi: 10.1242/bio.20134614 (PMC3744077; doi:10.1242/bio.20134614)
Supplement: Supplementary Material [file supp_bio.20134614_bio.20134614-s1.pdf]

Biology Open

Supplementary Material

Robert Bensch et al. doi: 10.1242/bio.20134614

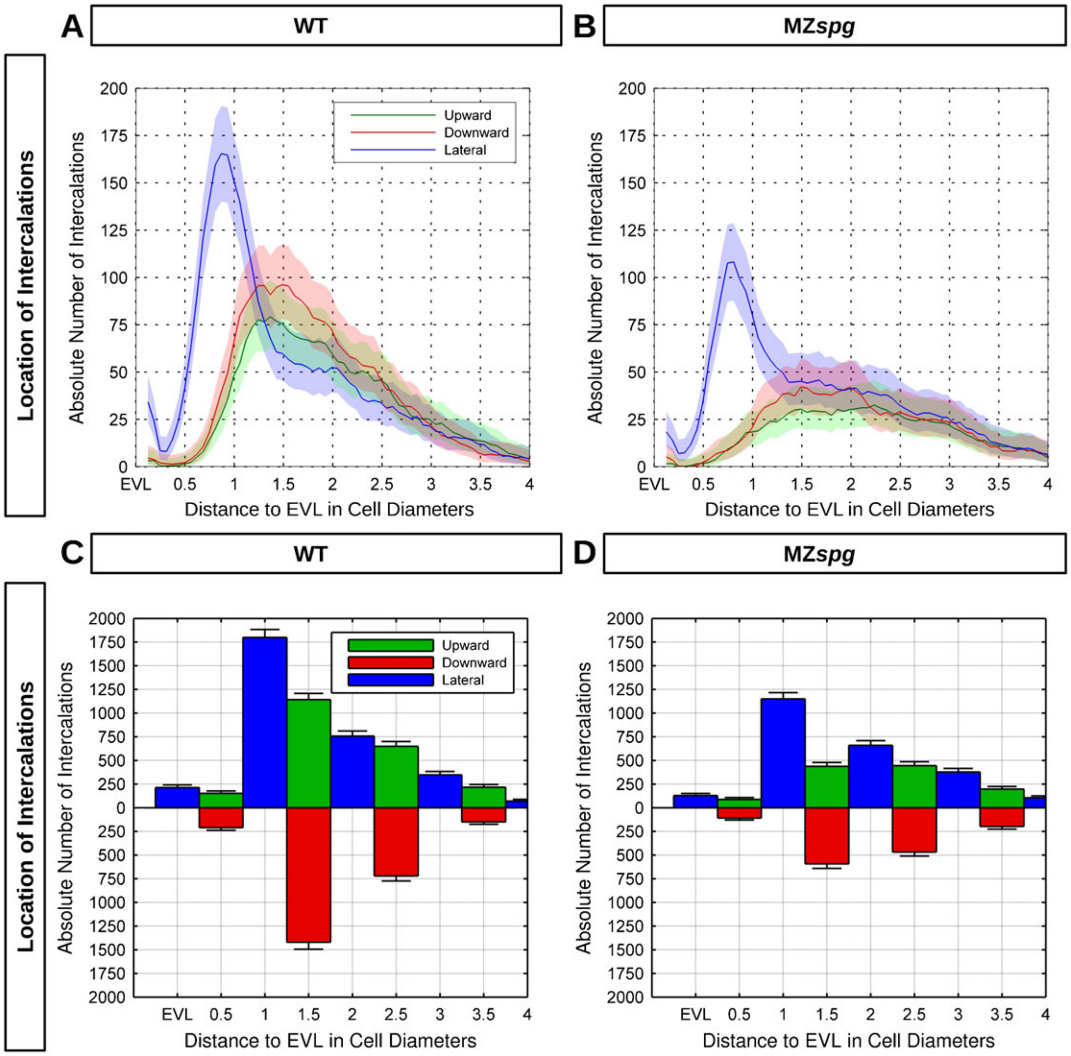

Fig. S1. Quantification of location of radial and lateral intercalation events. Quantification of WT (**A**,**C**) and MZ*spg* (**B**,**D**) blastomeres performing upward (green), downward (red), or lateralward (blue) intercalations in each depth level. Depth levels were numbered and distance was measured starting from the EVL at the animal pole towards the vegetal pole of the embryos. Compared to Fig. 3D,E the absolute (non-normalized) number of intercalations is shown (summed over all datasets). Besides the continuous representation (A,B) measurements are given discretized into bins of one cell diameter size (C,D) centered on integer distances to EVL for lateralward and in between for radial intercalations (n=6 embryos each for WT and MZ*spg*). The x-axis is truncated at 4.0, where the number of measured intercalations becomes too small to provide meaningful results. Errors are given by the 95% confidence intervals assuming Poisson noise.

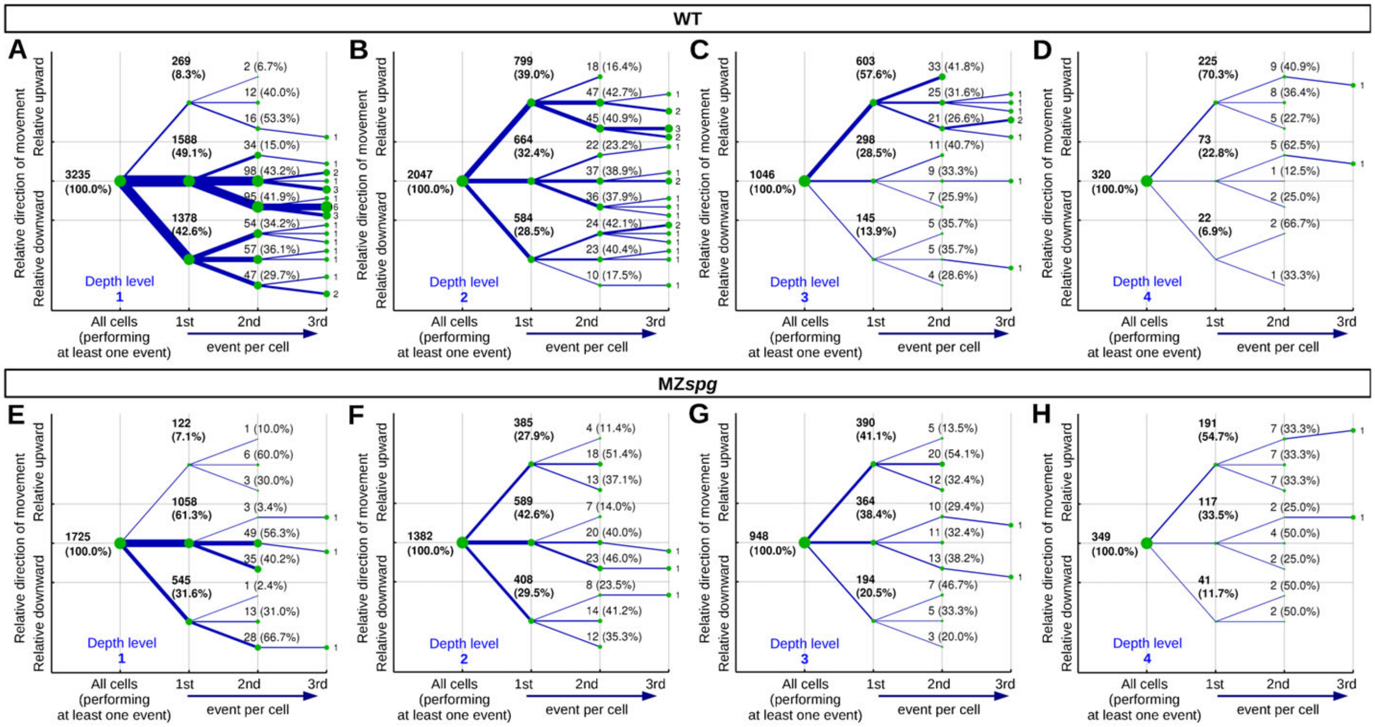

Fig. S2. Quantification of intercalation history of radial and lateral intercalation events. Summarized intercalation history of all individual cells for WT (**A–D**) and MZ*spg* (**E–H**) embryos (sum over six embryos for each genotype). The graph presents up to three successive intercalations of individual blastomeres, indicating upward, downward, or lateralward directions. The root node (leftmost) denotes all cells performing the first intercalation event. Compared to Fig. 3F,G the intercalation history is split according to the depth level where the first intercalation event is localized. From left to right the depth level increases by one. The absolute number and relative fraction of intercalations is given at each node.

Biology Open

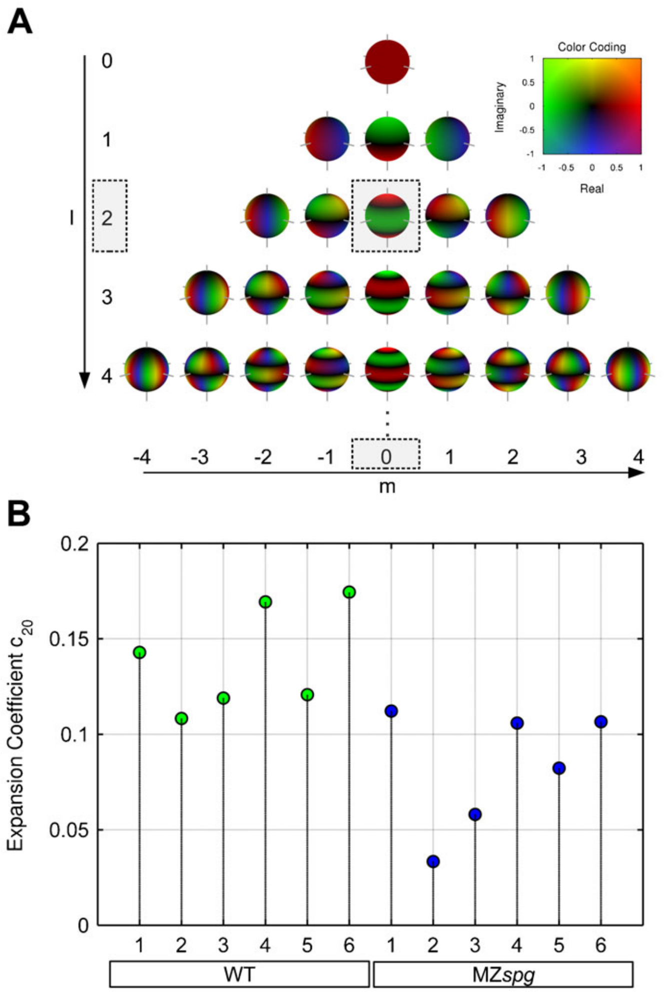

Fig. S3. Motion directionality of intercalation events: spherical harmonics and expansion coefficients c_{20}. (**A**) Spherical harmonics basis functions Y_{lm}, plotted for degree l=0…4 and order m=0…4. For the present application, especially Y_{20} (degree 2, order 0) is important. It represents signals that are polarized at the North and South pole. (**B**) Expansion coefficients c_{20} for all WT and MZ*spg* datasets representing the contribution of spherical harmonics basis function Y_{20} to their directionality distributions. Larger positive values represent stronger polarization at the North and South pole, smaller positive values represent weaker polarization respectively. WT shows significantly increased polarization compared to MZ*spg* embryos ($P<0.01$; n=6 embryos each for WT and MZ*spg*), see also Fig. 4.

Biology Open

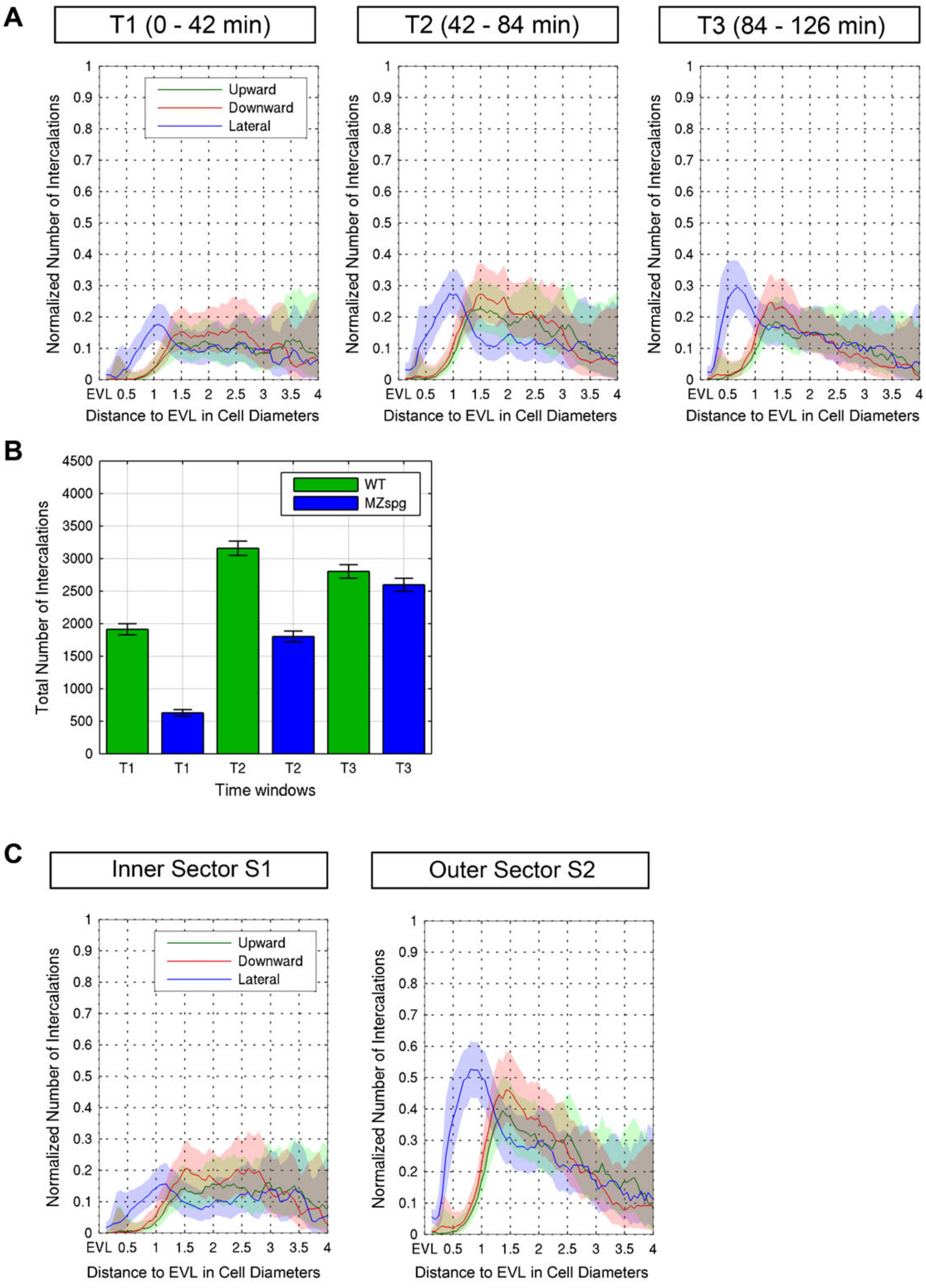

Fig. S4. Analysis of spatial and temporal patterns of intercalation in wildtype and MZ*spg*. (**A**) Quantification of WT blastomeres performing upward, downward, or lateralward intercalations in each depth level for the time windows T1 to T3. Depth levels as distance in average cell diameters were numbered and distance was measured starting from the EVL in vegetal direction. To be able to compare different depth levels, the absolute number of intercalations (summed over 6 embryos) was normalized by the total number of cells observed for each distance. The x-axis is truncated at 4.0, where the number of measured intercalations becomes too small to provide meaningful results. (**B**) Quantification of the total absolute number of intercalations for WT and MZ*spg* embryos in each of the three time windows T1 to T3 ($P<0.05$; summed over $n=6$ embryos each WT and MZ*spg*). Error bars show 95% confidence intervals assuming Poisson noise. (**C**) Quantification of WT blastomeres performing upward, downward, or lateralward intercalations in each depth level for the inner (S1) and outer (S2) sectors defined in Fig. 5. For details see panel A.

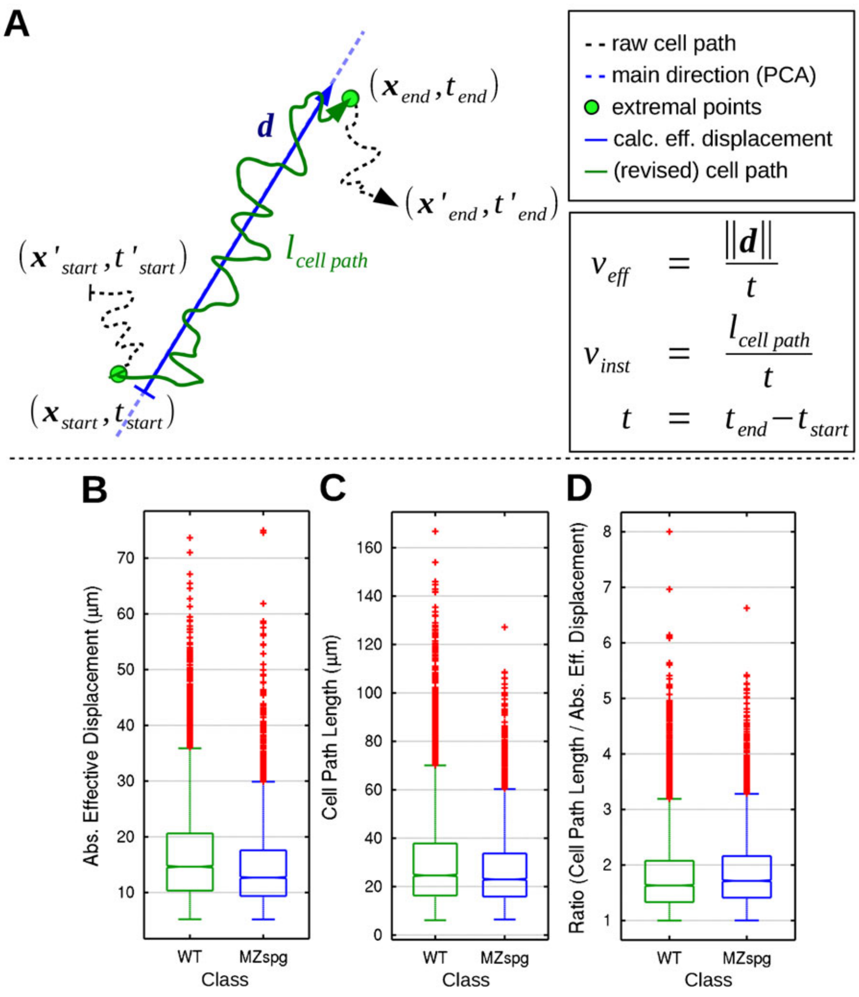

Fig. S5. Measurement of migration direction and path length of cells undergoing intercalation events. Radial intercalation dynamics. Complementary to Fig. 6, details about the measurement of migration direction and path length of blastomeres undergoing intercalation events are shown. (**A**) Schematic drawing of the raw cell path and the extracted features. (**B**,**C**) Complementary to Fig. 6B,C, the measured absolute effective displacements and cell path lengths are shown. (**D**) The ratios of cell path lengths (C) and absolute effective displacements (B) are shown. The ratio indicates how effective or directed cellular motion is during intercalation. A value of 1.0 indicates straight linear motion, while larger numbers indicate less effective or directed motion on a non-linear path.

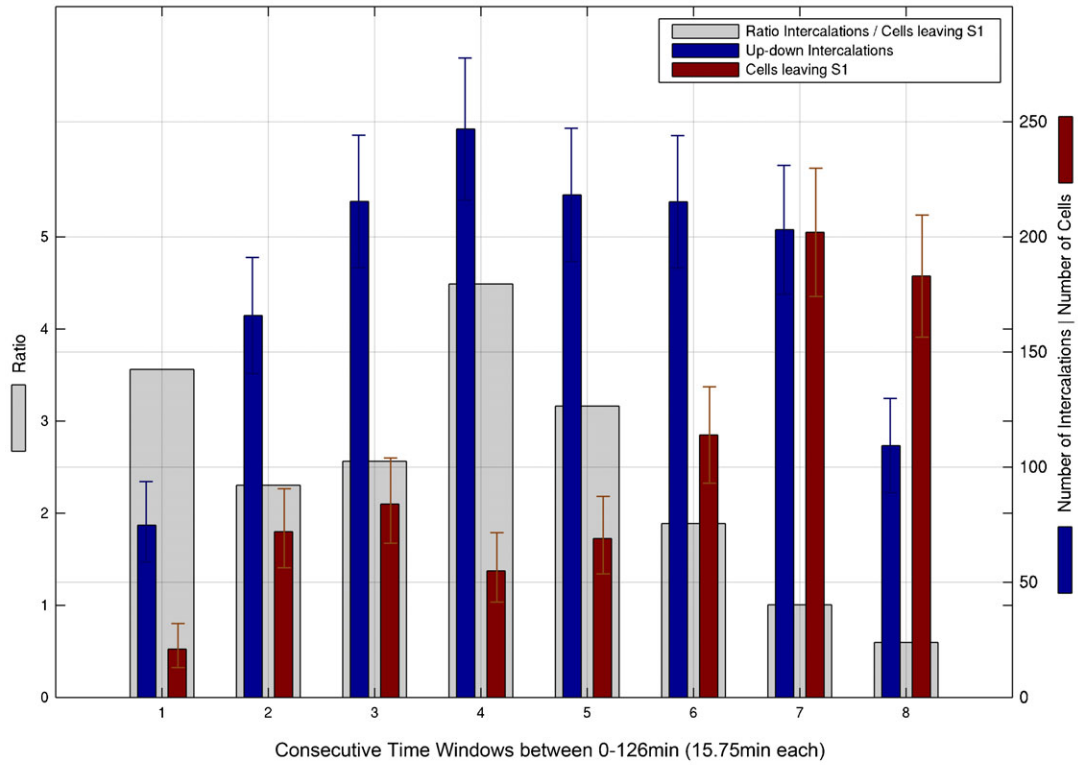

Fig. S6. Quantification of a potential contribution of radial intercalation to epibolic spreading in WT embryos. To determine whether the number of intercalations correlates with the number of cells leaving the inner sector S1 during shorter time windows, we defined eight consecutive time windows 1 to 8 (15.75 min each during the total observation time of 126 min), and quantified for each time window the number of radial intercalations and the number of cells leaving sector S1 (summed over n=6 embryos). The number of summed upward and downward intercalations in sector S1 (blue bars) is compared to the number of cells leaving sector S1 (brown bars). The ratio between both quantities is plotted in gray bars. Error bars show 95% confidence intervals assuming Poisson noise.

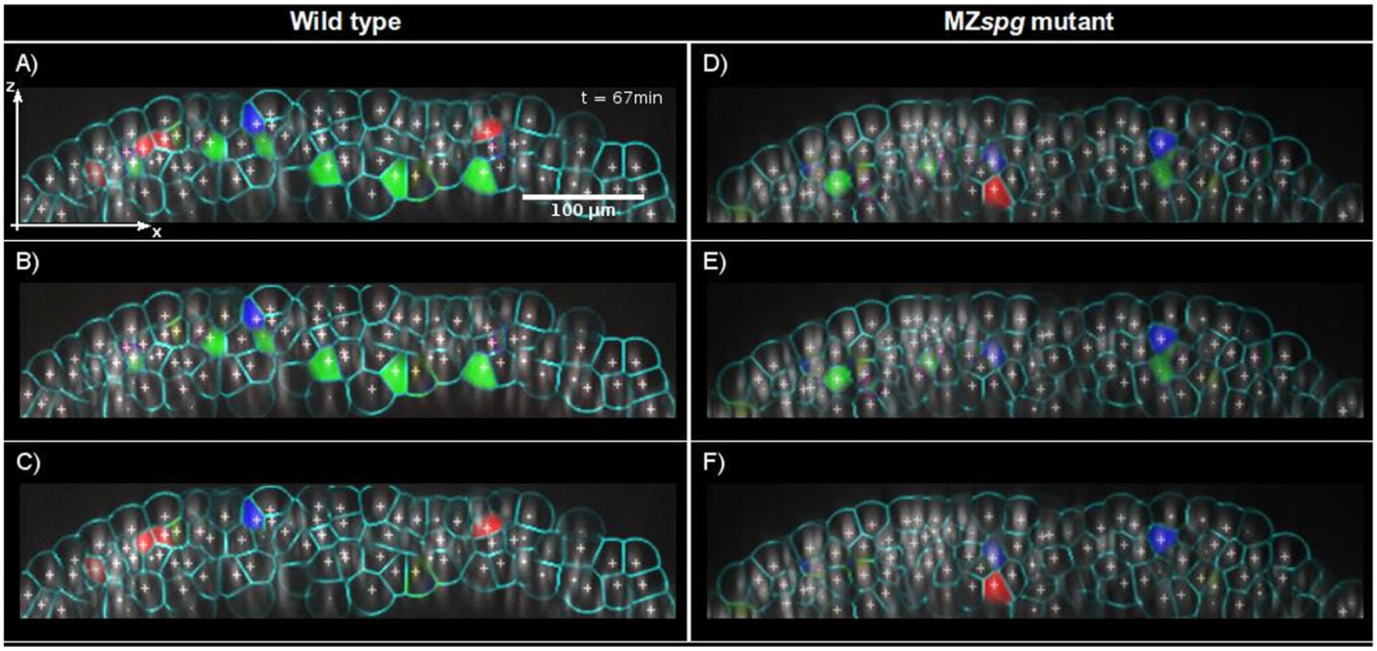

Movie 1. Radial intercalation events during blastomere movement. Global blastomere migration analyzed by computational detection and classification of radial intercalation events from two hour 3D time-lapse recording of WT (**A–C**) and MZ*spg* (**D–F**) embryos. Embryo stages: sphere to 50% epiboly. Confocal stacks (109 µm) were recorded from animal pole EVL into the margin of blastoderm over the doming yolk of the embryos. The renderings show lateral views (animal pole to the top) with raw nuclei fluorescence (grey), tracked nuclei positions (crosses) and calculated cell boundaries (cyan). Upward intercalations into more exterior levels (green), downward intercalations into more interior levels (red), and lateralward intra-level intercalations (blue) were detected along an 18 µm thick animal–vegetal oriented sheet transecting the embryo along its dorsoventral axis. (A,D) All intercalation events. (B,E) Upward and lateralward intercalations. (C,F) Downward and lateralward intercalations. Scale bar: 100 µm.

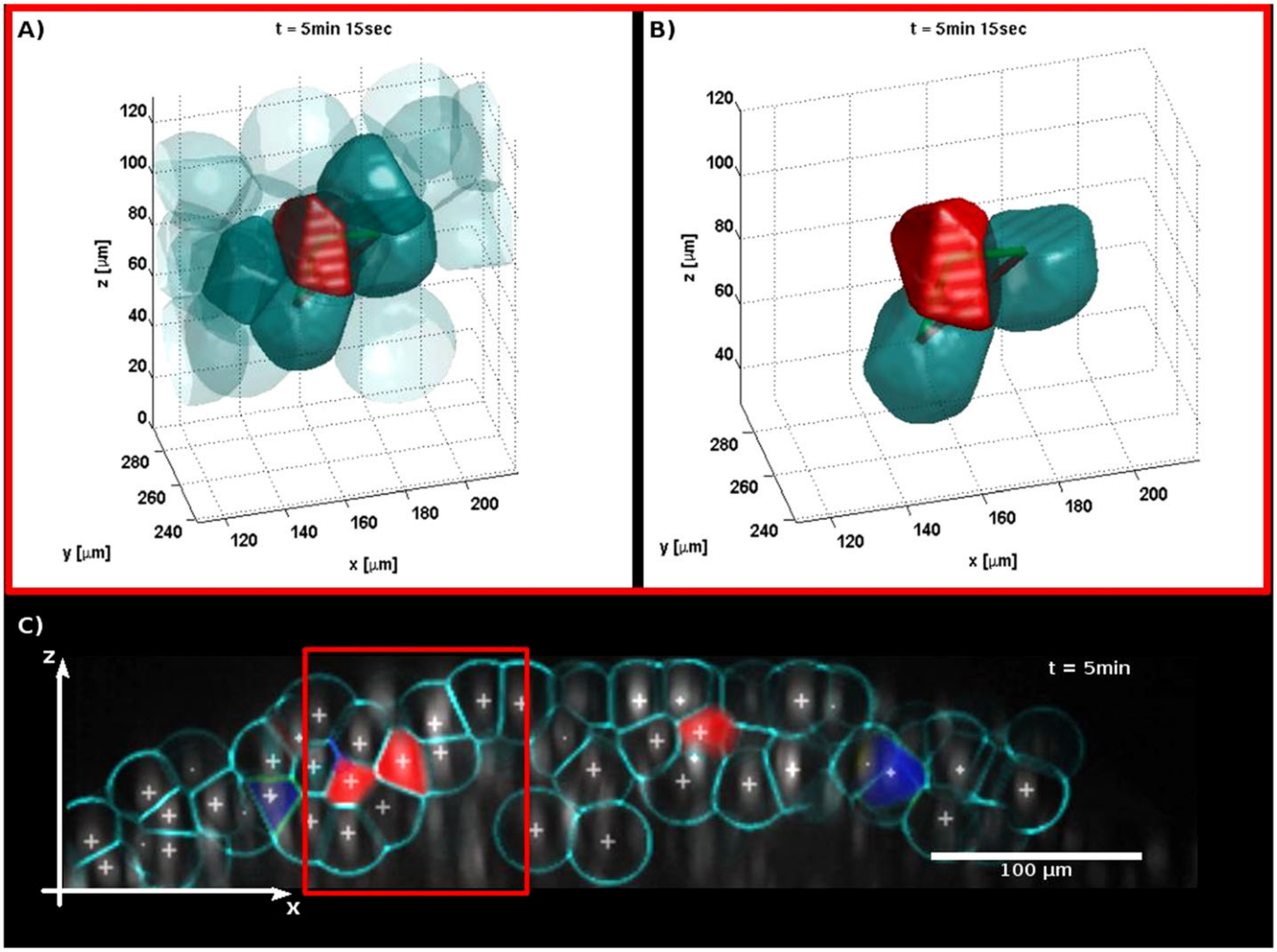

Movie 2. Examples for intercalation events within a group of blastomeres. Rendering of individual upward and downward intercalation events from 3D time-lapse recording of WT embryo (supplementary material Movie 1A–C). (**C**) Lateral view rendering (animal pole to the top) with raw nuclei fluorescence (grey), tracked nuclei positions (crosses) and calculated cell boundaries (cyan). Upward intercalations into more exterior levels (green), downward intercalations into more interior levels (red), and lateralward intra-level intercalations (blue) were detected along an 18 µm thick animal–vegetal oriented sheet transecting the embryo along its dorsoventral axis. Downward intercalation is shown exemplarily inside the red rectangle during the first 10 minutes of the movie. Upward intercalation is shown exemplarily inside the green rectangle during the following 20 minutes of the movie. (**A**,**B**) 3D rendering of corresponding individual cells performing upward or downward intercalation within a group of blastomeres. Scale bar: 100 µm.

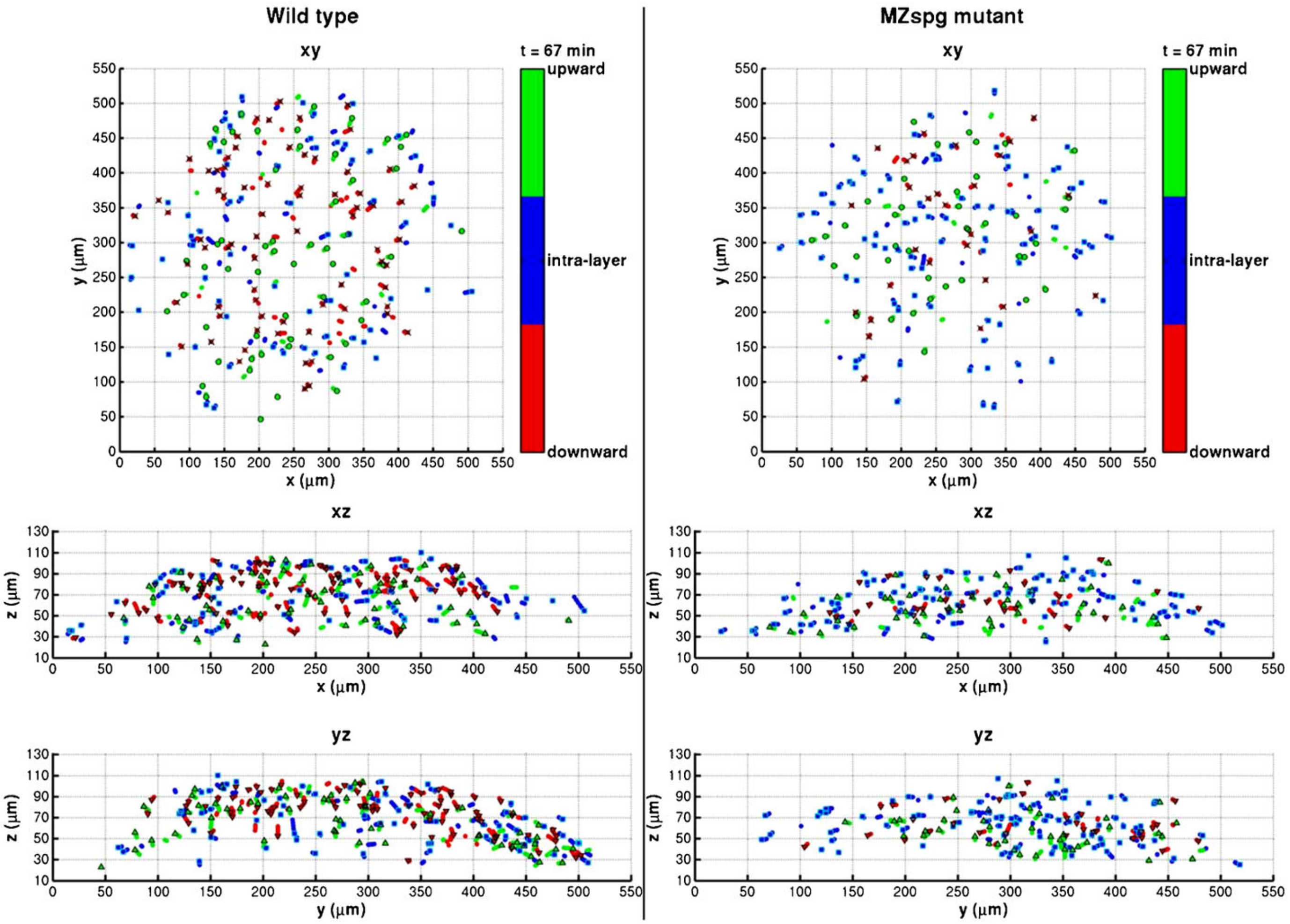

Movie 3. Directional analysis of migrating blastomeres. The calculated effective displacement (Fig. 6A) of all detected intercalations from WT and MZ*spg* embryos from two hour 3D time-lapse recording (supplementary material Movie 1) were visualized. Colors indicate the classified direction (upward: green, downward: red, and lateralward: blue). Three different views are shown: x–y plane (animal top view of the embryo), x–z plane and y–z plane (lateral views of the embryo). Motion direction is indicated in each view additionally by different symbols (lateralward: square; top view upward: circle, downward: cross; side views up-/downward: up-/downward pointing triangle). Point traces are shown for the past 5 minutes preceding each frame only.
